# Making progress in early breast cancer: Taking time or accepting risk?

**DOI:** 10.1016/j.ejca.2017.03.005

**Published:** 2017-06

**Authors:** Gurdeep S. Mannu, David Dodwell

**Affiliations:** a Clinical Trial Service Unit, Nuffield Department of Population Health, University of Oxford, United Kingdom; b Nuffield Department of Population Health, University of Oxford, United Kingdom

**Keywords:** Breast cancer, Clinical trials, Randomised trials, Observational studies, Surrogate markers

## Abstract

Outcomes for patients diagnosed with early breast cancer in developed countries have improved substantially over recent decades. Adjuvant therapies have contributed to this improvement and their benefits have been confirmed in large randomised controlled trials (RCTs) and meta-analyses. Lower event rates, whilst welcome, have created problems for RCTs, that need to be larger and often take longer to provide a reliable result. In an effort to maintain the rate of progress and to obviate the complexity, cost and time required to conduct large RCTs, there has been an increased tendency to rely on observational data to determine a treatment effect and also to accelerate progress by the use of a surrogate marker (pathological complete remission after neoadjuvant chemotherapy). We highlight the pitfalls in these approaches and suggest some simplifications in the conduct of RCTs.

## Highlights

• Surrogate markers are yet to be developed and validated in early breast cancer.
• Reliance on observational data to evaluate treatment effects is risky.
• Prospective randomised trials are difficult but remain vital to progress.
• Research approval processes need simplification and streamlining.

## 1 Introduction

Frustration at the slow rate of progress in trying to develop and improve treatments for early breast cancer is commonly expressed by both patient and medical communities. There is an obvious tension between the desire for improved treatments and other technologies and the need for careful evaluation. The pathway of progress in the management of this common disease is littered with examples of promising therapies, implemented prematurely and ultimately discarded when shown to be ineffective or unsafe.

## 2 Randomized prospective evidence

Although large treatment effects may in some circumstances be detected without prospective studies or with small trials, such revolutionary advances are very rare and there are none in the management of early breast cancer. In this disease there is a critical need to distinguish reliably between a moderate but worthwhile gain from a new treatment and no worthwhile gain. To achieve this, large-scale randomised evidence, interpreted without data-dependent emphasis on particular trials or subgroups, is needed. The randomised controlled trial (RCT) is accepted as the most reliable means of providing robust evidence of the efficacy and safety of new treatments, adaptations or withdrawal of existing treatments and new pathways of care.

Individual patient level meta-analysis represents the ultimate refinement of the biostatistical approach of the collection and analysis of prospectively collected, randomised data and offers greater statistical certainty of effect and may allow the safer exploration of a treatment effect in defined subgroups.

Unfortunately, deriving a result from a single RCT is costly and time-consuming. There are considerable bureaucratic, financial and often perceived ethical hurdles to be overcome before recruitment in a multicentre study can begin and these have worsened over time.

The prognosis for newly diagnosed patients with early breast cancer has improved and in particular screen-detected disease, with its generally much better prognosis, has become increasingly common. ‘Event rates’ have correspondingly fallen but this welcome development has meant larger clinical trials with longer follow up are required to reach a statistically secure endpoint. There is thus a substantial price to pay in time, cost and the number of trial participants needed, for a robust result that can improve care. A recent example is the EORTC trial of regional radiotherapy. This critically important trial included 4004 patients and demonstrated a 3% absolute (11% proportional) improvement in disease-free survival. This study was published 11 years after the last patient was recruited and 20 years since the trial started [1].

The long wait for results from RCTs is in contrast to the expressed desire for rapid progress and commercially problematic given the limited patent of newly licensed pharmaceuticals and the life span of other technologies. Academic success is also highly dependent on publication in high-profile journals and the long period of time between the awarding of funding for a RCT and the publication of results poses serious problems for academic researchers and their institutions.

The colossal expense, uncertainty over what should represent ‘best standard care’, large size, increasing emphasis on research governance, burgeoning bureaucracy and apparent slow progress related to the conduct of prospective clinical trials has tended to encourage the research community, funders and policy-makers to seek cheaper and quicker alternatives.

## 3 Surrogate markers

The use of surrogate markers is one potential approach to try to accelerate progress. An endpoint that can be derived relatively quickly (and preferably inexpensively) will (it is hoped) be predictive of longer-term benefit and allow earlier conclusions to be derived. This is an active area of clinical research and there are many differing approaches, but at this point in time none are in routine use and their validity in the form of formal testing against clinically established methods of assessment is at an early stage.

Perhaps the most significant development in the development of surrogate markers in early breast cancer is represented by the use of pathological complete response (pCR) as a possible indicator of future longer-term outcome following the use of neoadjuvant chemotherapy (NACT). The CTNeoBC study pooled information from 12 RCTs of NACT and confirmed an improvement in survival following the attainment of a pCR at individual patient level. Crucially this correlation could not be confirmed at clinical trial level limiting the ability to compare treatment approaches by a comparison of rates of pCR in groups of patients. In addition, the relationship between pCR and disease-free or overall survival at patient level was heavily influenced by biomolecular subtype—being much stronger for HER-2 positive or triple negative disease than for HER-2 negative, ER+ breast cancer [2].

Within the most recent meta-analysis of cytotoxic chemotherapy conducted by the EBCTCG adjuvant chemotherapy achieved the same proportional reduction in recurrence and mortality risk in ER positive (compared with ER negative) disease [3] despite the findings of much lower pCR rates when this treatment is used as neoadjuvant therapy [4]. The achievement of pCR clearly has prognostic significance but it seems less useful as a predictive marker of treatment benefit in most circumstances.

Although the US FDA agreed that the attainment of pCR following NACT could be used as an endpoint to support accelerated drug approval, there remains significant uncertainty of the usefulness and longer-term clinical validity of this approach [5], [6].

## 4 Observational data

Randomised evidence is not available to support the use of all interventions in the management of early breast cancer. Reconstructive surgery, newer diagnostic technologies, lymphoedema management and post-treatment surveillance imaging are interventions that are employed without supportive evidence of benefit from RCTs. Whether non-randomised evidence can be used to discern the benefits and toxicities of treatments with modest impacts on outcome, particularly commonly used adjuvant therapies, is highly contentious.

The advantages of observational evidence are the avoidance of the cost and time in setting up and running RCTs, the speed that results can be obtained and the ability to derive very large sample sizes. The limitations relate to the inability to predict all of the potential confounding variables that may affect the relationship between a selected treatment and its apparent therapeutic effects and toxicities and the difficulty in quantifying, and accounting for, the effect of confounding variables that can be predicted to exist.

In 1965, Austin Bradford Hill proposed nine criteria to help assess causality between an exposure such as cancer treatment and an outcome, such as cancer recurrence or cancer-related death or complication. These criteria included; strength of association, plausibility, biological gradient, consistency, specificity, temporality, coherence, experimental evidence and analogy with similar factors [7].

Whilst these criteria succinctly summarise the factors to consider in evaluating a potentially causal association they do not offer a definitive methodology to conclude causation. Over the 50 years since his publication, causation has been and is still commonly concluded by the fickle and variable nature of human judgement.

McGale *et al.* [8] reported worryingly divergent results between the effects of adjuvant radiotherapy on breast cancer mortality noted within the SEER database and those identified by the meta-analyses of RCTs evaluating the role of adjuvant radiotherapy conducted by the EBCTCG. These differences persisted despite detailed adjustment for potential confounding variables. SEER data also suggested that the risk of cardiac mortality was lower in patients receiving radiotherapy compared with those who did not receive this treatment, in contrast to data within EBCTCG that confirms that radiotherapy does slightly but significantly increase cardiac mortality.

Sagara *et al.* [9] also used SEER data to derive a prognostic score based on tumour size, patient age and nuclear grade for 32,144 patients with DCIS treated with breast conserving surgery. Their conclusion that higher risk patients derive a breast cancer and overall mortality benefit from the use of whole-breast radiotherapy is in marked contrast to the randomised evidence from the five individual trials of breast radiotherapy after excision of DCIS and the meta-analysis conducted by the EBCTCG, that suggest no impact on breast cancer–related mortality from the use of radiotherapy [10].

Perhaps the most prominent example of a treatment mistakenly introduced and used widely in high-risk early breast cancer is myelo-ablative chemotherapy with bone marrow rescue. On the basis of retrospective evidence, small uncontrolled studies and a widespread medical belief in superiority, many thousands of women were subjected to a risky and toxic treatment that, following the publication of several RCTs, is now known to be no or only slightly more effective, but unacceptably more toxic, than standard care [11].

Observational data have a place in the assessment of population-level outcomes following treatment (generalisability), the assessment of longer-term toxicity and can be of use when the characteristics of the population treated vary considerably from the participants of RCTs. To determine the presence and size of a modest (but worthwhile) treatment effect requires elimination of the insidious and underappreciated effects of bias. Other than in the very unusual circumstance of a treatment with a dramatic effect on outcome, such observational data are unhelpful in determining whether adjuvant treatments are more effective than standard care.

There are some circumstances where RCTs are not feasible and RCTs have on occasions—because of insufficient size or the over-interpretation of subgroups—provided spurious results.

However, the use of surrogate markers and the assessment of modest treatment effects by the acquisition of observational data at this point in time are not a substitute for prospective studies and randomised evidence designed to identify the presence of worthwhile benefits of new treatments. There is no immediate prospect of any change in this position.

The last 2–3 decades have seen substantial improvements in breast cancer mortality, falling local recurrence rates and more recently, reduced treatment-related morbidity. Improved systemic adjuvant therapies, the introduction of mammographic screening and refinements to locoregional treatments have played the major part in these improvements and all have evolved and been introduced following careful assessment in large randomised trials.

Simplification, de-escalation of bureaucracy and streamlining of research approval processes to encourage simple large prospective trials should remain a priority. Such trials can have optional shorter-term translational components involving for instance radiological, molecular pathological or detailed toxicity assessments in efforts to try to identify reliable surrogate markers that may ultimately obviate the long wait for recurrence and mortality data, but the majority of centres could recruit patients who would have routine follow up care alone.

‘Lighter touch’ research governance processes to simplify participation, reduce costs and remove much burdensome bureaucracy could be implemented for RCTs where toxicity risks are lower, for instance in duration trials, involving drugs that have been in routine use for many years. In future it may also be possible to use routinely collected data in national registries or other data sets to provide endpoint determination provided the accuracy of this approach can be verified.

Undue reliance on observational data or the premature use of surrogate markers in the contemporary assessment of new treatments or pathways of care in the management of early breast cancer involves a significant risk of embracing ineffective treatments or discarding those that are useful.

## Ethics

Not applicable.

## Funding

Gurdeep S. Mannu received funding from the National Institute of Health Research Biomedical Centre (Oxford). David Dodwell is funded by the Nuffield Department of Population Health, University of Oxford. Financial support is provided by Cancer Research UK (grant no C8225/A21133), by a research contract to the University of Oxford under the Department of Health Policy Research Programme (Studies of Ionising Radiation and the Risk of Heart Disease, 091/0203) as well as core funding from Cancer Research UK, the Medical Research Council and the British Heart Foundation to the Oxford University Clinical Trial Service Unit (grant MC_U137686858).

## Disclosures

None.

## Conflict of interest statement

None declared.

## References

[1] Poortmans P.M., Collette S., Kirkove C., Van Limbergen E., Budach V., Struikmans H., For the EORTC Radiation Oncology and Breast Cancer Groups (2015). Internal mammary and medial supraclavicular irradiation in breast cancer. N Engl J Med.

[2] Cortazar P., Zhang L., Untch M., Mehta K., Costantino J.P., Wolmark N. (2014 Jul 18). Pathological complete response and long-term clinical benefit in breast cancer: the CTNeoBC pooled analysis. Lancet.

[3] Early Breast Cancer Trialists' Collaborative Group (EBCTCG) (2012 Feb 10). Comparisons between different polychemotherapy regimens for early breast cancer: meta-analyses of long-term outcome among 100,000 women in 123 randomised trials. Lancet.

[4] Purushotham A., Pinder S., Cariati M., Harries M., Goldhirsch A. (2010 Aug 1). Neoadjuvant chemotherapy: not the best option in estrogen receptor–positive, HER2-negative, invasive classical lobular carcinoma of the breast?. J Clin Oncol.

[5] Prowell T.M., Pazdur R. (2012 Jun 28). Pathological complete response and accelerated drug approval in early breast cancer. N Engl J Med.

[6] Rose B.S., Winer E.P., Mamon H.J. (2016 Aug 22). Perils of the pathologic complete response. J Clin Oncol.

[7] Hill A.B. (1965 May). The environment and disease: association or causation?. Proc R Soc Med.

[8] McGale P., Cutter D., Darby S.C., Henson K.E., Jagsi R., Taylor C.W. (2016 Jul). Can observational data replace randomized trials?. J Clin Oncol.

[9] Sagara Y., Freedman R.A., Vaz-Luis I., Mallory M.A., Wong S.M., Aydogan F. (2016 Apr 10). Patient prognostic score and associations with survival improvement offered by radiotherapy after breast-conserving surgery for ductal carcinoma in situ: a population-based longitudinal cohort study. J Clin Oncol.

[10] Early Breast Cancer Trialists' Collaborative Group (EBCTCG) (2009 Dec). Overview of the randomized trials of radiotherapy in ductal carcinoma in situ of the breast. J Natl Cancer Inst Monogr.

[11] Mello M.M., Brennan T.A. (2001 Sep 1). The controversy over high-dose chemotherapy with autologous bone marrow transplant for breast cancer. Health Aff.

